# Supplementary material for: Cerebral Biomarkers and Blood-Brain Barrier Integrity in Preeclampsia
Source: Cells. 2022 Feb 24;11(5):789. doi: 10.3390/cells11050789 (PMC8909006; doi:10.3390/cells11050789)
Supplement: Supplementary file 1 [file cells-11-00789-s001.zip › cells-1550961-supplementary.pdf]

Supplementary information

Cerebral Biomarkers and Blood-Brain Barrier Integrity in Preeclampsia

Therese Friis[1*], Anna-Karin Wikström[1], Jesenia Acurio[2,3], Jose Leon[2,3,4], Henrik Zetterberg[5,6,7,8,9], Kaj Blennow[5,6], Maria Nelander[1], Helena Åkerud[10], Helena Kaihola[10], Catherine Cluver[11], Felipe Troncoso[2], Pablo Torres-Vergara[3,12], Carlos Escudero[#2,3], Lina Bergman[#1,11,13]

1 Department of Women's and Children's Health, Uppsala University, Uppsala, Sweden

2 Vascular Physiology Laboratory, Department of Basic Sciences, Faculty of Basic Sciences, University of Bío-Bío, Chillán, Chile

3 Group of Research and Innovation in Vascular Health (GRIVAS Health), Chillán, Chile

4 Escuela de Enfermería, Facultad de Salud, Universidad Santo Tomás, Los Ángeles, Chile

5 Department of Psychiatry and Neurochemistry, Institute of Neuroscience and Physiology, the Sahlgrenska Academy at the University of Gothenburg, Mölndal, Sweden

6 Clinical Neurochemistry Laboratory, Sahlgrenska University Hospital, Mölndal, Sweden

7 Department of Neurodegenerative Disease, UCL Queen Square Institute of Neurology, Queen Square, London, UK

8 UK Dementia Research Institute at UCL, London, UK

9 Hong Kong Center for Neurodegenerative Diseases, Hong Kong, China

10 Department of Immunology, Genetics and Pathology, Uppsala University, Uppsala, Sweden

11 Department of Obstetrics and Gynecology, Stellenbosch University, Cape Town, South Africa

12 Department of Pharmacy, Faculty of Pharmacy, University of Concepcion, Chile

13 Department of Obstetrics and Gynecology, Gothenburg University, Gothenburg, Sweden

# Shared last authorship

* Correspondence: therese.friis@kbh.uu.se; Tel.: 0046-18-6116613

Figure S1. Reduced protein abundance of claudin-5 in cell membrane of brain endothelial cells exposed to plasma from women with preeclampsia.

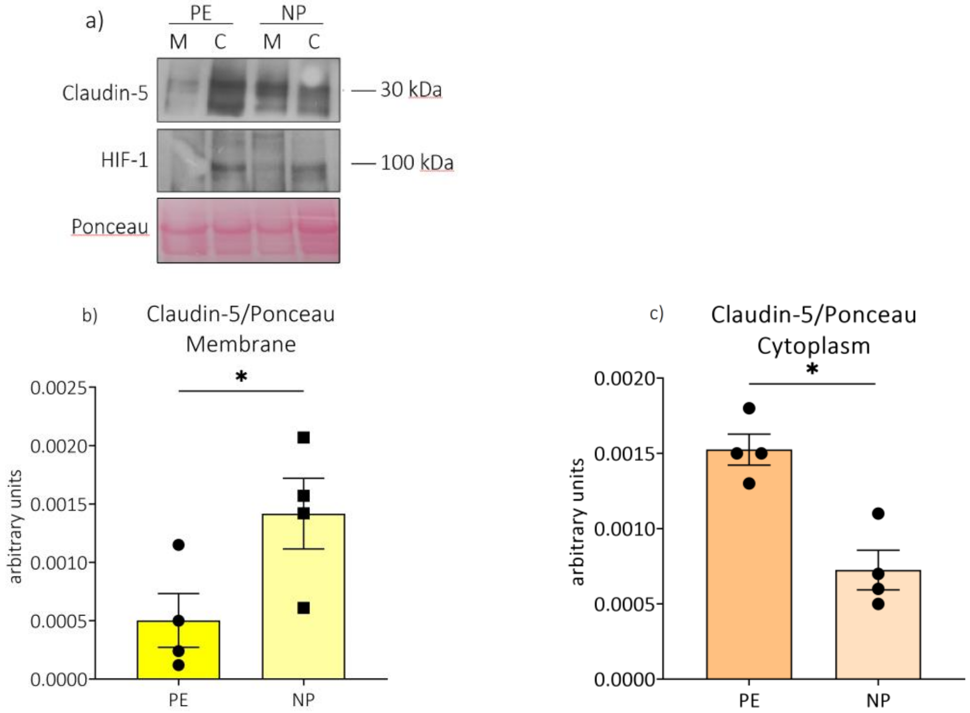

*Brain endothelial cell line hCMEC/D3 were incubated (12 h) with plasma from women with normal pregnancy (NP, n=4) or plasma from women with preeclampsia (PE, n=4). Plasma samples were randomly chosen from the whole group of 28 plasmas, respectively. After treatment, cells were used for extraction of membrane (M) and cytoplasmic (C) fractions by centrifugation. Briefly, hCMEC/D3 cells grown in 100 mm plates (maximum confluence) were washed with phosphate buffer solution (PBS, pH 7.4) and extracted with 100 µl of buffer lysis (Sigma-Aldrich, MO, USA) enriched with 1X protease inhibitors (Thermo Fisher Scientific, New York, USA). The lysate obtained was vortexed and homogenized constantly with the help of a 32G (0.23mm) syringe for approximately 15 times, and centrifuged at 2.000 rpm x 10 minutes at 4 °C. The supernatant obtained was again centrifuged at 14.000 rpm x 30 minutes at 4 °C, where a new pellet (membrane fraction) and supernatant (cytoplasm fraction) were obtained. The membrane fraction pellet was hydrated in lysis buffer, and joint with the cytoplasmic fraction they were stored at -20 °C until further analysis. For claudin-5 identification (key tight junction protein of the BBB), 50 µg of protein from the membrane and cytoplasm fractions were separated using SDS-PAGE (10%), transferred to nitrocellulose membranes, and probed with the primary antibody for claudin-5 (Abcam, Cambridge, UK; ab15106, dilution 1:1500 v/v). Rabbit (Thermo Scientific) secondary antibody conjugated with horseradish peroxidase was used for visualization. Identification of hypoxia inducible factor (HIF, Santa Cruz, CA, USA; sc-10790, dilution 1:2000 v/v) was used as negative control of membrane fractions, while Ponceau staining was used as loading control. Bands on gels were scanned and images quantified using ImageJ V1.48 software (National Institute of Health, USA) as previously described [1]. (a) Representative blot of analyzed proteins. (b) Densitometry of claudin-5/Ponceau ratio in the cell membrane fraction; or in (c) cytoplasmatic fraction. *p<0.05*

References

1. Escudero, C.; Bertoglia, P.; Hernadez, M.; Celis, C.; Gonzalez, M.; Aguayo, C.; Acurio, J. Impaired A2A adenosine receptor/nitric oxide/VEGF signaling pathway in fetal endothelium during late- and early-onset preeclampsia. *Purinergic Signalling* **2013**, *9*, 215-226, doi:10.1007/s11302-012-9341-4.
